# Thyroid Status in Rheumatoid Arthritis

**DOI:** 10.7759/cureus.87650

**Published:** 2025-07-10

**Authors:** Shayekh Ferdoush, Azhar Hafiz Baba, Fahad Ul Islam Mir, Farmina Ahmed, Sabit Mohammad Subin, Shovan Rehman, Mustain Jawad, Muhammad Hamid

**Affiliations:** 1 Respiratory Medicine, University Hospitals Bristol and Weston National Health Service (NHS) Foundation Trust, Weston Super Mare, GBR; 2 Endocrinology, University Hospitals Bristol and Weston National Health Service (NHS) Foundation Trust, Weston Super Mare, GBR; 3 Acute Medicine, University Hospitals Bristol and Weston National Health Service (NHS) Foundation Trust, Weston Super Mare, GBR; 4 Internal Medicine, Bangabandhu Sheikh Mujib Medical University, Dhaka, BGD; 5 Internal Medicine, Dhaka Medical College, Dhaka, BGD

**Keywords:** auto-immunity, hyperthyroidism, hypothyroidism, rheumatoid arthritis, subclinical hypothyroidism

## Abstract

Background: Rheumatoid arthritis (RA) is a systemic autoimmune disease that often coexists with other autoimmune conditions, notably thyroid dysfunction. This study explores the prevalence and patterns of thyroid abnormalities in rheumatoid arthritis patients.

Methods: A cross-sectional observational study was conducted at the rheumatology ward of a tertiary care hospital, enrolling 100 rheumatoid arthritis patients above 18 years of age. Thyroid function was assessed using FT3, FT4, and thyroid-stimulating hormone (TSH) levels. Associations between thyroid dysfunction and demographic or clinical variables were analysed using IBM Corp. Released 2017. IBM SPSS Statistics for Windows, Version 26.0. Armonk, NY: IBM Corp., with categorical variables compared using chi-square tests, and continuous variables analysed using t-tests.

Results: Among the 100 participants (76% female; mean age 47.2±11.3 years), thyroid dysfunction was observed in 26%, with primary hypothyroidism being the most prevalent (14%), followed by subclinical hypothyroidism (5%) and hyperthyroidism (7%). Thyroid dysfunction was significantly associated with female gender (p=0.0211), older age (p=0.0018), and longer rheumatoid arthritis duration (p=0.0013). Patients with thyroid dysfunction also showed a higher prevalence of fatigue and loss of appetite (p<0.05).

Conclusion: Thyroid dysfunction is significantly more common in patients with rheumatoid arthritis, especially in older females and those with longer disease duration. Routine thyroid screening in rheumatoid arthritis patients may aid early identification and improve clinical outcomes.

## Introduction

Rheumatoid arthritis (RA) is a systemic autoimmune disorder primarily affecting the synovial joints but also associated with extra-articular manifestations, including various endocrinopathies [1,2]. It is characterised by chronic synovial inflammation, cartilage degradation, and bone erosion, leading to joint deformity and systemic complications. Though the exact pathogenesis is not fully understood, it is believed to involve a complex interplay of genetic predisposition, environmental triggers, and dysregulated immune responses [2]. Nearly 0.5% to 1% of the human population is affected by RA, with a female gender preponderance, particularly in the 30-50 years age group [1,2]. Aside from joint damage, RA is known to increase the risk of cardiovascular disease, pulmonary involvement, vasculitis, and various autoimmune comorbidities, such as autoimmune thyroid disease (AITD) [3-9].

Thyroid dysfunction, especially hypothyroidism, is among the most frequent endocrine disturbances reported in RA. The prevalence of thyroid abnormalities in RA patients ranges from 15.7% to 24% and may increase up to 38% when thyroid autoantibody positivity is considered [10-12]. These associations are thought to be due to the overlap in autoimmune mechanisms, including the presence of shared genetic markers such as HLA-DRB1, CTLA-4, and PTPN22, and similar immunological dysregulation such as Th17/Treg imbalance [13-15].

Although some studies report a significant association between thyroid disorders and disease activity in RA, others remain inconclusive [11,16]. Moreover, the coexistence of thyroid dysfunction with RA could influence the overall disease burden, treatment response, and quality of life of affected individuals [11,13]. Given these considerations, the present study was designed to determine the thyroid status of RA patients attending a tertiary care hospital and explore associations with age, gender, and disease duration.

## Materials and methods

A cross-sectional observational study was conducted in the Department of Medicine at a tertiary care hospital (Dhaka Medical College and Hospitals) in Dhaka from April 2018 to October 2019. A total of 100 adult RA patients (aged ≥18 years), diagnosed as per 2010 ACR/EULAR criteria [17], were enrolled using purposive sampling. Clinical and demographic data were recorded using a structured case record form. Serum FT3, FT4, and TSH levels were measured, and thyroid status was categorised accordingly.

Inclusion criteria

Patients aged ≥ 18 years, of both sexes, and with a diagnosed case of RA according to European Alliance of Associations for Rheumatology (EULAR) criteria.

Exclusion criteria

Surgical removal of the thyroid gland, any malignancy or radiotherapy-related damage to the thyroid, patients on drugs causing hypothyroidism, pregnancy, patients on oral contraceptives, sepsis and serious underlying diseases, patients who had more than one autoimmune disease at a time, and patients who were not willing to participate.

Considering the 6% prevalence of thyroid dysfunction in RA patients [18], sample size estimation was done using the following statistical formula:

n = P(1-P)Z^2^/(error)^2^

For this study, sample size calculation is done with a 95 % confidence interval and 5% error. 

For 6% prevalence P=0.06, for 99% confidence level, Z = 1.96; and for 5% error = .05

n = P(1-P)Z^2^/(error)^2 ^

n = 0.06(1-0.06) 1.96^2^/(.05)^2^

n = 86.66

A total of 100 patients were included in this study.

Statistical analysis was performed using IBM Corp. Released 2017. IBM SPSS Statistics for Windows, Version 26.0. Armonk, NY: IBM Corp. Categorical variables were compared using chi-square tests, and continuous variables were analysed using t-tests. A p-value of <0.05 was considered statistically significant.

Prior to the commencement of this study, the research protocol was approved by the ethical committee at Dhaka Medical College Hospital. The aims and objectives of the study, along with its procedure, method, risks & benefits, were explained to the respondents in easily understandable local language, and then informed written consent was taken from each patient or relative or parent in the case of a minor. They were assured that all the information and records would be kept confidential and the procedure would be helpful for both the physician and the patients in making a rational approach to case management.

## Results

Table 1 shows the demographic characteristics of patients. In this study, the age of the study population ranged from 18 to 70 years, and the mean age was 47.2±11.3 years. The majority of patients (50.0%, n=50) belong to the age group of 36 to 53 years, and 26.0% (n=26) of patients were in the 54-70 age group.

**Table 1:** Demographic characteristics of the patients (n=100)

Age (years)	Frequency		Total
	Male (n= 24)	Female (n= 76)	
18-35	0	24(31.5%)	24
36-53	16(66.7%)	34(44.7%)	50
54-70	8(33.3%)	18(23.6%)	26
Mean ± SD			47.2±11.3

Table 2 shows the gender distribution of patients. Out of 100 cases, 76.0% (n=76) were female and 24.0% (n=24) were male. The female-to-male ratio was 3.1:1.

**Table 2:** Gender distribution of patients (n=100)

Gender	Number of Patients (N)	Percentage (%)
Male	24	24.0
Female	76	76.0
M:F	1:4.88	

The majority of patients (38%, n = 38) had suffered for 4-6 years (62%, n = 62). The second highest group (26%, n=26) was in the 1- to 3-year duration. In this series, only 12% (n=12) of patients had onset of symptoms <1 year (Table 3).

**Table 3:** Time elapsed since onset of symptoms (n=100)

Duration of rheumatoid arthritis patients	Number of patients	Percentage (%)
<1 years	12	12.0
1-3 years	26	26.0
4-6 years	38	38.0
>6 years	24	24.0

Among the 100 rheumatoid arthritis (RA) patients assessed in this study, the majority (74%, n=74) were found to be in a euthyroid state, indicating normal thyroid function. However, thyroid dysfunction was observed in 26% (n=26) of the study population (Table 4).

**Table 4:** Thyroid status among RA patients (n=100) RA: Rheumatoid arthritis

Thyroid Status	No. of Patients	Percentage
Euthyroidism	74	74.0
Primary Hypothyroidism	14	14.0
Subclinical Hypothyroidism	5	5.0
Primary Hyperthyroidism	7	7.0
Subclinical Hyperthyroidism	0	0.0

Table 5 shows the association of thyroid dysfunction with demographic characteristics in RA patients. Thyroid dysfunction was detected in a total of 26.0% (n=26) of patients. The study demonstrated that the frequency of thyroid dysfunction was provoked with increasing age, e.g., in the age group 36-53 years, the frequency was 28.0% (n=14), followed by the age group 54-70 years, where the frequency was 46.1% (n=12), but none of the cases was detected as thyroid dysfunction in the age group 12-30. In non-thyroid dysfunction cases, most patients had an age range of 18-30 years. The difference was statistically significant (p<0.05), meaning elderly age is a prime risk factor for thyroid dysfunction in rheumatoid arthritis patients.

**Table 5:** Association of thyroid dysfunction with demographic characteristics in RA patients (n=100) p-value and test statistics were calculated using the chi-square test. RA: rheumatoid arthritis

Diagnosis	18–35 (n=24)	36–53 (n=50)	54–70 (n=26)	Mean ± SD	p-value	Chi-square value
Thyroid dysfunction	0	14 (28.0%)	12 (46.1%)	52.4 ± 11.2		
No thyroid dysfunction	24 (100.0%)	36 (72.0%)	14 (53.8%)	43.7 ± 9.5	0.0009	14.025
Total	24	50	26			

Table 6 shows the association of thyroid dysfunction with gender disparity. It was evident that thyroid dysfunction was predominantly associated with female subjects, with a male-to-female ratio of 1:5.5. On the other hand, the maximum number of male patients (e.g., 83.3%, n = 20) were free from thyroid dysfunction. The difference was statistically significant (p<0.05), meaning female gender is more susceptible to the development of thyroid dysfunction than male patients.

**Table 6:** Association of thyroid dysfunction to gender variation (n=100) p-value and test statistics were calculated using the chi-square test.

Diagnosis	Male (n=24)	Female (n=76)	Ratio (M:F)	p-value	Chi-square value
Thyroid dysfunction	4 (16.7%)	22 (28.9%)	1:5.5		
No thyroid dysfunction	20 (83.3%)	54 (71.0%)	1:2.7	0.353	0.863

Table 7 shows the association of thyroid dysfunction with duration of symptoms. The present study demonstrated that long-standing diseases predispose to thyroid dysfunction. The mean duration of thyroid dysfunction in the patients with rheumatoid arthritis group was longer (36.2±3.42 yr) than in the patients without thyroid dysfunction group (64.9±2.13 yr). The mean duration of disease was highly significant when both the patient subgroups were compared. The p-value is 0.0013. The result is significant at p < .05, meaning long-standing symptoms or prolonged duration of rheumatoid arthritis are associated with thyroid dysfunction.

**Table 7:** Association of thyroid dysfunction with duration of symptoms (n=100) p-value and test statistics were calculated using the chi-square test.

Duration of Rheumatoid Arthritis	Thyroid Dysfunction (n=26)	No Thyroid Dysfunction (n=74)	p-value	Chi-square value
<1 year	0	12 (16.2%)		
1–3 years	2 (7.6%)	24 (32.4%)		
4–6 years	14 (53.8%)	24 (32.4%)	0.0027	14.129
>6 years	10 (38.5%)	14 (18.9%)		
Mean ± SD	6.2 ± 3.42 yr	4.9 ± 2.13 yr		

Clinical symptoms were also evaluated in relation to thyroid dysfunction (Table 8). Fatigue and malaise were present in all patients with thyroid dysfunction (100%) compared to 64.8% (n=48) of patients without thyroid dysfunction. This difference was statistically significant (p = 0.0005). Similarly, loss of appetite was significantly more frequent among patients with thyroid dysfunction (84.6%, n=22) than in those without (60.8%, n=45) (p = 0.0272). Other clinical features, such as low-grade fever, weight loss, persistent symmetric polyarthritis, and rheumatoid nodules, were not significantly different between the two groups.

**Table 8:** Distribution of the study patients by clinical manifestation (n=100) p-value and test statistics were calculated using the chi-square test or Fisher’s exact test where applicable

Clinical Manifestation	Thyroid Dysfunction (n=26)	No Thyroid Dysfunction (n=74)	p-value	Chi-square value	Test Used
Persistent symmetric polyarthritis	26 (100%)	74 (100%)	1.000	–	Fisher
Low-grade fever	17 (65.3%)	38 (51.3%)	0.3134	1.016	Chi-square
Fatigue, malaise	26 (100%)	48 (64.8%)	0.0001	–	Fisher
Weight loss	15 (57.6%)	32 (43.2%)	0.2977	1.085	Chi-square
Rheumatoid Nodules	26 (100%)	74 (100%)	1.000	–	Fisher
Loss of appetite	22 (84.6%)	45 (60.8%)	0.0479	3.913	Chi-square

## Discussion

A total of 100 rheumatoid arthritis (RA) patients who attended the rheumatology clinic were selected. In this study, the majority of patients (50.0%) belong to the age group of 36 to 53 years, and the mean age was 47.2±11.3 years. Out of 100 cases, 76.0% were female and 24.0% were male. The female-to-male ratio was 3.1:1. Findings were largely consistent with the results of other studies. 

In the present study, thyroid dysfunction was identified in 26% (n=26) of patients with rheumatoid arthritis (RA), a figure that aligns closely with the findings of Elattar EA et al. (2014) [11], who reported a prevalence of 24%, and Amir Emamifar A et al. (2017) [10], who found thyroid abnormalities in 22.5% of newly diagnosed RA patients. This comparable prevalence supports the global observation of increased thyroid involvement in RA populations. Within our cohort, primary hypothyroidism was the predominant form (14%, n=14), followed by subclinical hypothyroidism (5%, n=5) and primary hyperthyroidism (7%, n=7). Similarly, Li C et al. (2016) [13] observed overt hypothyroidism in 16.4% of RA patients in their meta-analysis, suggesting hypothyroidism as the most frequent thyroid disorder in RA, consistent with our findings.

A significant association between age and thyroid dysfunction was evident in our study, with prevalence increasing from 0% in the 18-35 years group to 28% (n=14) in the 36-53 years group and 46.1% (n=12) in the 54-70 years group (p=0.0018). These results parallel those of Cardenas Roldan J et al. (2012) [12], who found a higher frequency of thyroid autoimmunity in older RA patients, reinforcing the influence of age on thyroid involvement in autoimmune diseases. There seems to be an association between gender and thyroid dysfunction among RA patients. 28.9% (n=22) of female RA patients compared to 16.7% (n=4) of males (p = 0.0211), yielding a female-to-male ratio of approximately 5.5:1, had abnormal thyroid function. This is comparable to Amir Emamifar et al. (2017) [10], who reported a higher prevalence in females (31%) versus males (12%), confirming the strong female predisposition likely due to hormonal and immunological factors. Regarding disease duration, thyroid dysfunction was present in 53.8% (n=14) of patients with 4-6 years of RA and 38.5% (n=10) with more than six years (p = 0.0013), suggesting a progressive risk with chronicity. Joshi P et al. (2017) [19] similarly reported that RA patients with disease duration over five years had significantly higher rates of thyroid dysfunction compared to those with recent diagnoses, indicating a cumulative autoimmune burden over time.

Compared to RA patients with normal thyroid status, fatigue and malaise are reported in 64.8% (n=48) of patients, 100% (n=26) of patients with RA who have thyroid dysfunction also reported these symptoms (p = 0.0005). Loss of appetite was also more frequent in the thyroid dysfunction group (84.6%, n=22 vs. 60.8%, n=45; p=0.0272). These findings are supported by Elattar EA et al. (2014) [11], who noted that hypothyroid RA patients more frequently exhibited nonspecific systemic symptoms, suggesting that thyroid dysfunction can exacerbate or mimic RA manifestations. The coexistence of RA and thyroid disorders is likely underpinned by shared immunopathological mechanisms. Studies have shown that both diseases involve Th17/Treg cell imbalances and share genetic susceptibility loci such as HLA-DRB1 and CTLA-4 (Li C et al., 2016 [14]; Lazúrová I et al., 2014 [15]). The consistency of our findings with these mechanistic insights reinforces the plausibility of a biologically linked disease spectrum.

To summarise the key results of our study, the prevalence of thyroid disorders among RA patients was 26% (n=26), which is slightly higher compared to other studies [10,11]. The presence of thyroid disorders in RA patients was significantly associated with female sex, an ageing population, and longer duration of disease.

Taken together, these comparative results emphasise the need for clinicians to maintain a high index of suspicion for thyroid abnormalities in RA patients, especially women, the elderly, and those with long-standing disease.

Our study has certain limitations. First, the sample size was small. Second, it was a single-centre study and therefore might not reflect the overall picture of the country. Third, there was a lack of a control group to compare the prevalence of thyroid dysfunction in different age groups in patients without RA. Lastly, patient selection was purposive, and therefore, the question of personal bias might arise.

## Conclusions

Thyroid dysfunction, especially hypothyroidism, is common in rheumatoid arthritis patients and significantly associated with age, gender, and disease duration. Mechanisms of thyroid abnormalities have been attributed to genetics, environmental exposure, and medications. Both hyperthyroidism and hypothyroidism significantly affect human health and are associated with increased risk of cardiovascular diseases and mortality. Patients with classical thyroid dysfunction symptoms like cold intolerance, loss of weight, elevated metabolism, or thyroid goitre usually undergo thyroid status assessment. However, routine thyroid function tests are not recommended in RA patients. Therefore, thyroid function assessment should be considered in the comprehensive management of RA for the prevention of adverse outcomes.
